# Lead Poisoning in the Americas: Sources, Regulations, Health Impacts, and Molecular Mechanisms

**DOI:** 10.3390/jox15040134

**Published:** 2025-08-20

**Authors:** Blanca Miriam Torres-Mendoza, Asbiel Felipe Garibaldi-Ríos, Lourdes Del Carmen Rizo De La Torre, Ana María Puebla-Pérez, Luis E. Figuera, Guillermo Moisés Zúñiga-González, Belinda Claudia Gómez-Meda, Itzae Adonai Gutiérrez-Hurtado, Elvia Harumi Scott-López, Verónica Vázquez-González, Celeste Patricia Gazcón-Rivas, Martha Patricia Gallegos-Arreola

**Affiliations:** 1 Laboratorio de Inmunodeficiencias y Retrovirus Humanos, División de Neurociencias, Centro de Investigación Biomédica de Occidente, Instituto Mexicano del Seguro Social, Guadalajara 44340, Jalisco, Mexico; blanca.torresm@imss.gob.mx; 2 Departamento de Clínicas Médicas, Centro Universitarios de Ciencias de la Salud, Universidad de Guadalajara, Guadalajara 44340, Jalisco, Mexico; 3 Programa de Doctorado en Genética Humana, Centro Universitario de Ciencias de la Salud, Universidad de Guadalajara, Guadalajara 44340, Jalisco, Mexico; asbiel.garibaldi4757@alumnos.udg.mx (A.F.G.-R.); luisfiguera@yahoo.com (L.E.F.); 4 División de Genética, Centro de Investigación Biomédica de Occidente, Centro Médico Nacional de Occidente, Instituto Mexicano del Seguro Social, Guadalajara 44340, Jalisco, Mexico; celestegazcon@gmail.com; 5 División de Medicina Molecular, Centro de Investigación Biomédica de Occidente, Instituto Mexicano del Seguro Social, Guadalajara 44340, Jalisco, Mexico; lourdes.rdlt@gmail.com (L.D.C.R.D.L.T.); mutagenesis95@gmail.com (G.M.Z.-G.); 6 Laboratorio de Inmunofarmacología, Centro Universitario de Ciencias Exactas e Ingenierías, Universidad de Guadalajara (UdeG), Guadalajara 44430, Jalisco, Mexico; ana.puebla@academicos.udg.mx; 7 Departamento de Biología Molecular y Genómica, Instituto de Genética Humana “Dr. Enrique Corona Rivera”, Centro Universitario de Ciencias de la Salud, Universidad de Guadalajara, Guadalajara 44340, Jalisco, Mexico; belinda.gomez@academicos.udg.mx (B.C.G.-M.); itzae.gutierrez@academicos.udg.mx (I.A.G.-H.); 8 Laboratorio de Salud en el Trabajo, CMNO, IMSS, Guadalajara 44340, Jalisco, Mexico; elvia.scott@imss.gob.mx; 9 Unidad de Medicina Familiar 05 “Dr. Benito Kondo”, Instituto Mexicano del Seguro Social, El Salto 45680, Jalisco, Mexico; veronica.vazquezg@imss.gob.mx

**Keywords:** lead, lead poisoning, vulnerable population, lead regulation, blood lead levels

## Abstract

Lead poisoning is a significant public health issue, contributing to 0.6% of the global disease burden and disproportionately affecting developing countries. Vulnerable populations, such as children, pregnant women, and low-income communities, remain at high risk, often exposed to lead levels exceeding safe thresholds. While the problem is global, this review focuses specifically on the Americas, regions with diverse regulatory landscapes and persistent environmental lead exposure. Regulatory frameworks vary widely, and the lack of global consensus on acceptable blood lead levels leaves important gaps in protection. This review compiles and updates knowledge on emerging sources of lead exposure in the region, evaluates advancements in regulatory approaches, and analyzes the molecular impacts of lead on human health. Using the Comparative Toxicogenomics Database (CTD), lead was found to interact with 3448 genes, including those linked to inflammation and oxidative stress, and is associated with 4401 diseases and 799 disrupted pathways. These findings emphasize the need for regionally tailored interventions, strengthened policies, and further research on its health impacts.

## 1. Introduction

Lead is a contaminant metal that is widely distributed naturally in the Earth’s crust, is recognized as an environmental toxicant, and is highly toxic to humans due to its bioaccumulation capacity [1]. Lead poisoning accounts for 0.6% of the global burden of disease, a percentage that increases in developing countries [2,3]. It is not known to have a critical physiological role in the body, the human body does not metabolize it into other elements, and it is known that there are no safe blood lead levels [4,5]. Many cases of chronic or acute poisoning causing disease and disability are recognized by progressive accumulation of lead in the human body [6,7,8].

Factors that can influence the absorption of lead in the body are the size of the particles to which it is exposed, the chemical species, and its absorption through the digestive tract, respiratory, dermal and prenatal routes [2,4]. The internalization pathway of lead once inside the body is by its binding to erythrocytes; it then it passes into soft tissue, and eventually accumulates in the bones. The extent of lead intoxication depends on different factors such as dose, exposure time, and age at the beginning of exposure, among other factors [9,10]. The main known sources of intoxication are direct contact with soil, water, air, contaminated food, or everyday utensils containing or contaminated with lead [8,11].

According to the United Nations Children’s Fund (UNICEF) [12] about 800 million children worldwide have lead levels equal to or greater than 5 µg/dL and, according to World Health Organization (WHO) data [13], one million people die from poisoning [4].

Lead exposure is a problem that can affect all humans, especially vulnerable populations such as women of reproductive age and children, worldwide, with emphasis on low- and middle-income countries [14].

In this review, we present a synthesis of the sources, regulations, health impacts, and molecular mechanisms associated with lead poisoning, with a specific focus on the Americas. We begin by contextualizing the historical use of lead, highlighting how its widespread application in daily life persisted for centuries before its toxic effects on human health were fully understood. As scientific knowledge progressed, regulatory frameworks gradually evolved to address the risks of exposure. We also delve into the molecular mechanisms by analyzing the main genes that interact with lead and the biological pathways affected, aiming to understand how this metal alters key cellular processes such as inflammation, oxidative stress, and intracellular signaling. This review integrates multidisciplinary evidence to emphasize that lead remains a significant public health threat in the region, while underscoring the need to refocus attention on vulnerable populations, update regulatory standards, and advance research on its molecular effects.

## 2. Historical Context

**Figure 1:**
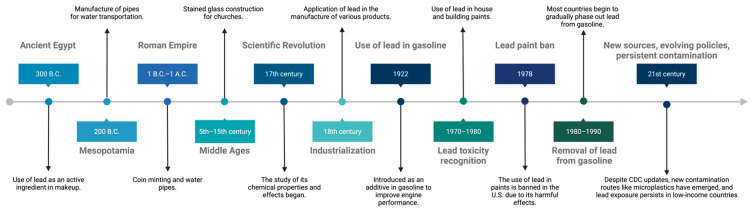
Timeline of the historical context of lead use. Created in BioRender. Garibaldi, A. (2025) https://BioRender.com/nhz1kku.

Lead has been used by humans for over 6000 years, due to its physicochemical properties that make it versatile and inexpensive (Figure 1). Ancient civilizations employed it for the manufacture of various utensils, tools and decorative objects [1,5,15]. Over time, the attractiveness of its benefits has justified its use, even when its negative health implications were known.

A significant period of lead exposure was in its use as an additive in gasoline, which peaked between 1960 and 1980. During the 1970s, lead levels in gasoline began to decline substantially in the United States, resulting in a decrease in lead exposure in children [16]. However, the effects of this prolonged exposure were already evident. In 1994, lead poisoning was considered the most common environmental disease in the United States, affecting mainly the development of erythrocytes, kidneys, and the nervous system, with consequences such as delayed neuronal development, decreased intelligence, and behavioral alterations [17].

Prior to 1994, the main form of poisoning in adults was by inhalation in occupational settings, while in children, exposure was mainly associated with ingestion of lead from the environment (water, soil, paint, ceramics, and medicines, among others). At the same time, lead-containing paint was identified as an important risk factor, as it was used in homes, buildings and other structures, and was released into the environment over time, due to wear and tear [17].

According to the Agency for Toxic Substances and Disease Registry (ATSDR), blood lead levels decreased significantly between 1960 and 2012 [18]. The elimination of lead in gasoline was a breakthrough in reducing poisoning. This change, along with other health interventions, has led to a significant decrease in blood lead levels in the global population. Recent studies in European and North American countries report blood lead levels below 1 μg/dL, while in South America blood lead levels are below 3 μg/dL [19].

In 2012, the U.S. National Toxicology Program concluded that there was sufficient evidence of adverse health effects in children and adults at blood lead levels below 5 µg/dL (ATSDR, 2024). It is now recognized that there are no safe blood lead levels, and the threshold that was thought to be tolerable for the body has changed over time. In 1985, the limit was set at 60 μg/dL, reducing to 25 μg/dL in 1991, to 10 μg/dL in 2012, and finally to 5 μg/dL [2].

In addition to regulatory developments, social, racial, socioeconomic, and environmental factors play a crucial role in the unequal distribution of lead exposure. Urban communities, particularly those with fewer resources, are the most affected, where it negatively impacts the health and quality of life of these populations, being more severe in developing countries. Limited access to quality drinking water, education, and personal hygiene products, as well as the use of clean fuels, are determining factors in the health of the most vulnerable children and communities in these contexts [20,21].

The history of the 20th century shows a growing concern for urban industrial lead contamination, especially in soil, which remains a significant legacy source of lead concentration in urban areas [22].

In the 21st century, new challenges and advancements related to lead exposure and regulation have emerged. One notable development is the revision of blood lead reference levels by the U.S. Centers for Disease Control and Prevention (CDC), which lowered the threshold for children from 5 µg/dL to 3.5 µg/dL in 2021 [23], acknowledging the risks of even minimal exposure. Additionally, novel environmental sources have been identified, such as microplastics, which can adsorb and transport lead through water, soil, and food systems [24]. Persistent contamination in urban and industrial settings remains a concern, particularly in low- and middle-income countries, where regulatory enforcement may be weak [25,26]. These 21st-century developments underscore the ongoing evolution of lead-related risks and the need for updated public health responses and regulatory frameworks.

In Latin America, despite global progress, lead exposure remains a significant public health issue. Several countries still face challenges related to historical contamination, limited regulation, and insufficient surveillance. For example, in Mexico, the use of traditional lead-glazed pottery continues to be a source of chronic exposure, especially in rural communities and marginalized urban areas [27,28]. In Peru, La Oroya has been classified among the most polluted cities in the world, due to decades of smelting activities, with documented elevated blood lead levels in children living near the metallurgical complex [29,30]. Similarly, Uruguay faced a national lead poisoning crisis in La Teja, a neighborhood affected by informal battery recycling and industrial waste [31,32].

Moreover, countries like Brazil and Argentina have reported persistent lead contamination associated with mining and informal industrial activities [33,34,35]. These examples reflect the unequal environmental burden faced by vulnerable populations in the region, often compounded by poverty, lack of access to clean water, and poor housing conditions.

The historical use of lead reflects a longstanding human reliance on its physical and chemical advantages, often at the expense of health. Despite early awareness of its harmful effects, lead continued to be widely used until well into the 20th century, with gasoline additives and lead-based paints representing major sources of exposure. Regulatory interventions, such as the removal of lead from gasoline and the ban on lead paints, have led to a significant decline in blood lead levels, globally. However, the persistence of lead in soils, older infrastructure, and emerging sources like microplastics underscores the fact that lead exposure remains a relevant public health concern, particularly in underserved and vulnerable populations.

## 3. Regulations and Strategies for Lead Poisoning Prevention

Lead poisoning is a global public health problem, although regulatory efforts to manage it have advanced significantly in recent decades. In the United States, lead regulation began in the 1970s, with key federal legislation such as the Safe Drinking Water Act of 1974, the lead Pollution Control Act of 1988, and the Lead and Copper Rule of 1991. More recently, the Lead in Drinking Water Reduction Act of 2011 consolidated efforts to minimize exposure at critical sources [36].

Internationally, there are no uniform standards for the prevention and management of lead poisoning, although many countries have adopted national protocols based on the U.S. Center for Disease Control and Prevention (CDC) guidelines, which set the reference level for blood lead levels exposure in adults at 5 μg/dL, reducing it to 3.5 μg/dL for children as of October 2021, after analyzing the results of the National Health and Nutrition Examination Survey [37].

Policies in industrialized countries have begun to focus not only on detection of hazardous levels of lead in the environment, but also on primary prevention. However, many studies suggest the need to further reduce blood lead levels considered safe, with proposals to lower detection thresholds from 10 μg/dL to 2 μg/dL [5].

Although advances in the elimination of blood lead levels in gasoline have significantly reduced exposure levels, regulation in other contexts, such as drinking water, remains crucial. According to the WHO [13], the recommended level of lead in drinking water is 10 parts per billion, a threshold that has been adopted by several countries as a safety standard [36].

Regulation varies in different regions of the world, leading to inequalities in lead exposure. In the United States, it has been observed that low-income neighborhoods, where racialized migrant communities predominate, show higher blood lead levels [16]. This inequality highlights the importance of including environmental and socioeconomic factors in public health strategies to address lead exposure, as suggested by recent studies [20].

In Latin America, lead is one of the most studied pollutants, although research has decreased since 2005, limiting adequate monitoring and preventive actions [21]. The lack of updated data on blood lead levels in children in the region underscores the need to strengthen research and public policies to reduce the knowledge gap and promote environmental health [38]. Furthermore, in many Latin American countries, lack of economic resources and lack of health education limit the capacities of families to mitigate risks from lead exposure [39].

New public policies aimed at primary prevention and remediation of lead effects in vulnerable communities are required. Strengthening health surveillance systems and promoting international partnerships to improve cooperation in the fight against lead contamination are essential to advance global public health protection [22,40]

Based on the above, we consider it necessary to address the unequal exposure to lead through fair and evidence-based public policies. First, it is proposed that governments locally implement mandatory blood-lead screening programs for children and pregnant women in high-risk areas, given that even low levels can affect child neurodevelopment and maternal health [41,42,43]. Second, international organizations such as the WHO and PAHO should harmonize global reference levels for blood lead, updating them according to evidence demonstrating toxicity at concentrations previously considered safe [44,45]. Third, there is an urgent need to invest in infrastructure to replace drinking water systems that contain lead, particularly in marginalized communities, as has been documented in prior studies [46,47,48]. Finally, it is advisable to implement environmental justice frameworks that incorporate socioeconomic data into risk assessments and prioritize remediation efforts in vulnerable populations, as recommended in other approaches aimed at protecting disadvantaged communities [49,50].

Despite advances in lead regulation, significant disparities persist globally, due to the lack of uniform standards and the influence of socioeconomic and environmental factors. Strengthening surveillance, updating reference levels, and investing in infrastructure are key actions to reduce exposure, especially in vulnerable populations.

## 4. Diversity of Lead Poisoning Sources

**Figure 2:**
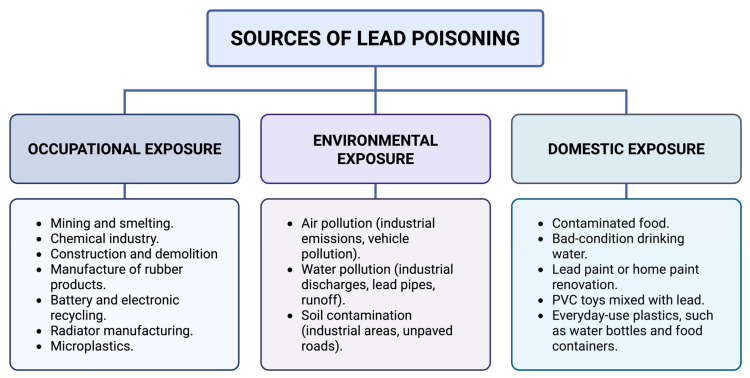
Sources of lead poisoning. Lead exposure occurs primarily through occupational, environmental, and domestic routes. Each pathway involves specific sources, such as industrial activities, environmental pollution, and household items [1,6,53,54,55]. Created in BioRender. Garibaldi, A. (2025) https://BioRender.com/2237o8i.

There are several sources of lead contamination, which can be classified as occupational, environmental and domestic (Figure 2). Occupational exposure is recognized as the main source of poisoning, especially in sectors such as mining, the chemical industry, smelting, construction, rubber product manufacturing, lead battery recycling, and radiator manufacturing. In mining areas, people living in their vicinity often have higher-than- average lead levels, with occupations related to mineral extraction and smelting being mainly responsible for this phenomenon [9].

Ingestion of lead-contaminated food is one of the most significant routes of exposure, since lead, like other heavy metals, does not break down or degrade, accumulating along the food chain [51]. Mining and smelting, together with the use of batteries and fossil fuels, are the main sources of environmental contamination by lead, affecting both air and water, and living in rural areas, in conditions of poverty or malnutrition, increases vulnerability to environmental exposure [52,53].

The daily use of certain products also contributes to the risk of exposure. Common examples are worn metal pipes, traditional herbal medicine, recent renovation of household paint, and proximity to areas with high vehicular traffic. Living in dwellings with dirt floors or near unpaved roads and houses with old pipes are factors that increase blood lead levels [3,6,11,20]. As for domestic sources, lead is also found in PVC toys for children, particularly in developing countries [1]. In addition, lead has also been reported in varying concentrations in everyday-use plastics, such as water bottles, food containers, toys, and packaging materials, representing an additional source of environmental and domestic exposure [54,55,56,57].

In the Americas, the sources of lead contamination are diverse and less well known and often overlooked or underestimated. Industrial facilities, mines, and toxic-waste dumps contribute to exposure, along with environmental factors that facilitate contamination, such as lack of regulation, and social problems such as poverty and income inequality [14,38]. These situations require multifaceted approaches that address both local factors and global causes of pollution. Activities such as electronics recycling, especially in low-resource regions in Latin America, represent an important source of exposure, often involving children in these practices [58]. The use of lead in glazed ceramics in Mexico is a relevant source of poisoning in certain communities. In Latin America and the Caribbean, other sources have been documented, such as informal battery recycling and other informal activities in Uruguay [28,58] lead-contaminated soil in urban zones of Argentina and Brazil [59,60], and residual pollution in Peru and Chile [29,61,62]. In Jamaica, elevated blood lead levels have been reported in children living near busy streets and those who consume certain fruits [63].

Recent studies have pointed out that climatic conditions, such as increasing land-surface temperature, can influence the release of lead from pipes and mobilization of lead in soil dust, especially during the summer and autumn seasons; this increases exposure in urban areas [22].

It is important to highlight that, in addition to classical sources of lead exposure, recent studies have revealed the presence of heavy metals such as lead in microplastics, which represent an emerging route of both environmental and human exposure. These plastic fragments, commonly found in water, soil, and food, have the ability to adsorb or contain toxic metals, acting as carriers that facilitate their transport and bioaccumulation in living organisms [24,64,65]. This scenario underscores how lead contamination has evolved beyond traditional sources, adapting to modern environmental contexts and posing new challenges for public health. The widespread distribution of microplastics and their capacity to adsorb hazardous substances on their surface, such as heavy metals and persistent organic pollutants, amplify the potential risks, particularly in vulnerable populations and ecosystems already burdened by pollution.

As previously observed, lead exposure arises from a wide range of occupational, environmental, and domestic sources. While traditional sources like mining and paint remain relevant, emerging vectors such as plastics and microplastics highlight the evolving nature of lead contamination. These findings underscore the need for integrated strategies to address both known and emerging risks, particularly in vulnerable populations.

## 5. Lead Toxicokinetics and Mechanisms

**Figure 3:**
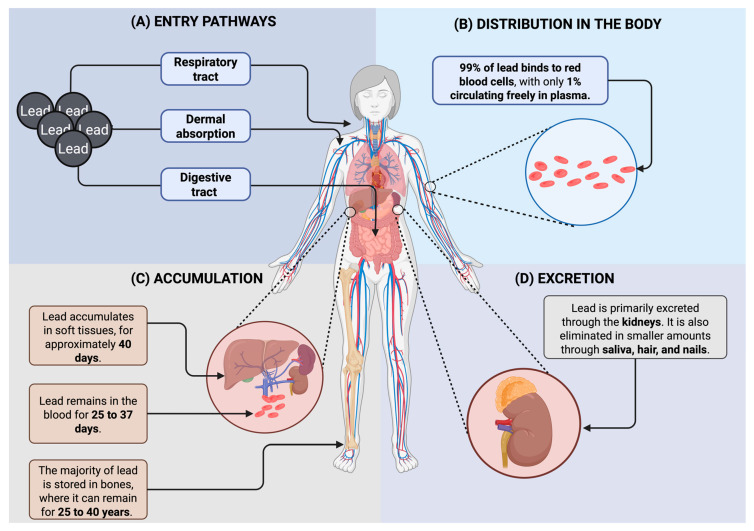
Absorption, distribution, accumulation, and excretion of lead in the human body. (**A**) Lead enters through the respiratory and digestive tracts, as well as via dermal contact. (**B**) Once absorbed, it circulates, mostly bound to red blood cells. (**C**) Lead accumulates in blood, soft tissues, and bones, with varying retention times. (**D**) Excretion occurs primarily through the kidneys, with minor elimination via other routes [5,67]. Created in BioRender. Garibaldi, A. (2025) https://BioRender.com/1jl8wtf.

Lead can enter the body mainly through the digestive or respiratory tract, and it can also enter through the dermal and prenatal routes [4]. Once absorbed, approximately 99% of lead binds to red blood cells, while only 1% remains in plasma. It is then distributed to various compartments, including the blood, soft tissues (particularly the liver and kidneys), and bones. Lead remains in the bloodstream for about 25 to 37 days, accumulates in soft tissues for approximately 40 days, and can persist in bones for 25 to 40 years, where it acts as a long-term reservoir. In adults, up to 90% of the total body burden of lead is stored in bones and teeth, compared to about 70% in children. The main route of excretion is renal, although smaller amounts can be eliminated through saliva, hair, and nails [5,66] (Figure 3).

As previously observed, lead enters the body through multiple routes, and accumulates in blood, soft tissues, and bones, where it can persist for decades. Its clinical manifestations are often nonspecific, making diagnosis difficult, but primarily involve neurological, renal, and gastrointestinal systems. These effects underscore the importance of early detection and long-term monitoring to prevent irreversible health consequences.

## 6. Health Impacts of Lead Poisoning on Pregnant Women, Children and Adults

### 6.1. Impact of Lead Exposure on Maternal and Fetal Health During Pregnancy

Lead contamination negatively affects the reproductive health of women, and, during pregnancy, can affect both maternal health and embryo development, and pregnancy outcomes [3,68]. Several studies have demonstrated the consequences for the health of pregnant women and the fetus, due to the presence of lead blood levels (Table 1).

### 6.2. Effects of Lead Poisoning in Children

Children are the most vulnerable to the effects of lead, followed by pregnant women [3,9]. Lead is known for its negative effects on neuronal development, causing cognitive deficits, emotional dysregulation, learning difficulties, self-control problems, and behavioral disturbances. Even low levels of exposure can be reflected in adulthood, in aspects such as educational achievement, criminal behavior and physical health [16,70]. Children are at greater risk due to age-typical behaviors such as constantly putting their hands in their mouths and handling objects that may be in contact with contaminated surfaces, as well as spending more time on the ground and playing outdoors [67]. Once lead is consumed through contaminated foods, children can absorb between 40–50% of the oral dose of lead [51]. Elevated levels of lead in maternal serum have also been associated with malformations, infant death, and decreased brain development [66]. This metal interferes with the mental and motor development of children, affecting their cognitive and emotional growth [11].

In studies, it was observed that lead levels in children under 5 years of age were higher. This is attributed to their behavior, since repeated contact of the mouth with the hands increases the exposure of the mucous membranes to lead, which increases its absorption [11]. The results of these studies coincide with others that indicate that exposure to lead above 3.3 μg/dL is associated with a decrease in the development of language skills [52].

The nervous system is particularly vulnerable to lead poisoning, especially during the early stages of life [10]. Elevated levels of lead blood levels in childhood have been associated with lower structural integrity of the brain and decreased cognitive function in middle adulthood, as observed in studies using magnetic resonance imaging [71]. Furthermore, exposure to lead during neurological development can trigger irreversible alterations in the structure and function of the central nervous system [10].

Refugee children constitute an even more vulnerable population, due to anemia and malnutrition, factors that increase lead absorption [67]. These children, along with other groups in disadvantaged socioeconomic situations, face additional risk due to their increased exposure to contaminated environments. Scientific evidence links early exposure to lead with adverse neurological and cognitive outcomes, such as reduced brain volume, decreased IQ, poorer working memory and processing speed, and impaired perceptual reasoning [72]. Furthermore, exposure to lead during childhood is closely related to community factors such as poverty level and housing age. In areas with higher poverty rates and older housing, children are at higher risk of being in contact with materials containing lead, such as paint and pipes [73].

### 6.3. Lead Poisoning in Adults

In adults, lead absorption varies between 3% and 10% when consumed through contaminated foods. At the population level, exposure to this heavy metal can generate a series of adverse health effects, including anemia, nephropathy, hypertension and infertility [11]. In addition, adults who were exposed to lead in childhood may present persistent problems related to behavior, hearing, speech, cognitive impairment, depression, personality disorders, anxiety, and an increased risk of hypertension and cardiovascular diseases [8,71].

There are studies that have shown an association between greater exposure to lead and a higher probability of premature death, although the exact mechanisms through which lead contributes to this higher mortality are not yet fully clarified [74].

Adults who were exposed to lead during childhood experience alterations in several systems, particularly in the brain, bones, and cardiovascular system. These alterations affect fine motor skills, emotional regulation, and cognitive ability, which can significantly influence the life trajectory of these individuals [75].

Millions of children who were exposed to high levels of lead in the past, due to the use of lead in gasoline, present sequelae that are reflected in behavioral dysregulation, affecting their ability to lead a full, successful, and healthy life, in adulthood [71].

As evidenced throughout this section, lead exposure causes profound health effects across all age groups, particularly in pregnant women and children. These impacts include impaired fetal development, neurodevelopmental disorders, and long-term cognitive and emotional deficits. In adults, both past and ongoing exposures are associated with chronic diseases, neurological decline, and reduced quality of life, emphasizing the importance of timely prevention and sustained public health efforts.

## 7. Molecular Mechanisms of Lead Toxicity

As previously described, lead exposure affects human health across all life stages, with particularly severe consequences in vulnerable populations such as children, pregnant women, and the elderly. Given the broad and complex nature of these health effects, one of the central objectives of our research was to explore the molecular mechanisms underlying lead-induced toxicity. To achieve this, we used the Comparative Toxicogenomics Database (CTD) [76], which allowed us to identify interactions between lead and key genes involved in fundamental cellular processes. This approach aims to deepen our understanding of how lead exerts its toxic effects at the molecular level, ultimately contributing to the identification of potential therapeutic targets or the development of evidence-based preventive strategies.

### 7.1. Top Genes Interacting with Lead

According to the CTD platform [77], lead interacts with 3448 genes, with the highest number of interactions reported for *TNF* (Tumor Necrosis Factor), *CYP1A1* (Cytochrome P450 Family 1 Subfamily A Member 1), and *CAT* (Catalase), all of which are key players in inflammation and oxidative stress. Other frequently affected genes include *MT1* and *MT2* (Metallothioneins 1 and 2), *ALAD* (Aminolevulinate Dehydratase), *CASP3* (Caspase-3), *HMOX1* (Heme Oxygenase 1), *APP* (Amyloid Beta Precursor Protein), and *NFE2L2* (Nuclear Factor, Erythroid 2 Like 2), suggesting their central role in the cellular response to lead.

**Figure 4:**
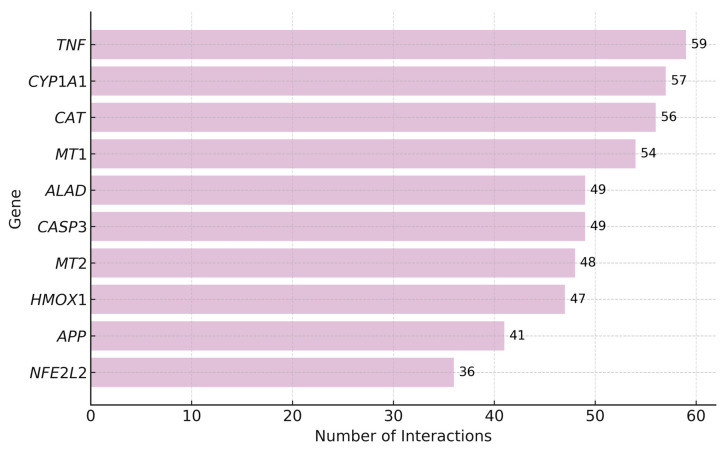
Top Genes with the Highest Number of Interactions with Lead. As shown in the figure, *TNF* has the highest number of interactions with lead (59), followed by *CYP1A1* (57), *CAT* (56), and *MT1* (54). Other genes such as *ALAD* and *CASP3* (49 each), *MT2* (48), *HMOX1* (47), *APP* (41), and *NFE2L2* (36) also exhibit a high number of interactions.

The following Figure 4 shows the top 10 genes with the highest number of interactions with lead.

*TNF* is a key inflammatory cytokine whose overexpression under lead exposure may promote chronic inflammation and tissue damage [77,78,79,80]. *CYP1A1* participates in xenobiotic metabolism, and its overactivation can increase reactive oxygen species (ROS), contributing to oxidative stress and genotoxicity [81,82,83,84]. *CAT*, an antioxidant enzyme, is impaired by lead, which reduces cellular defense against oxidative damage and promotes mitochondrial dysfunction and apoptosis [85,86,87,88].

Metallothioneins *MT1* and *MT2* regulate metal homeostasis and detoxification. Although their upregulation can bind lead and mitigate toxicity [89,90,91,92]. *ALAD*, essential for heme biosynthesis, is inhibited by lead, leading to impaired hemoglobin synthesis and accumulation of neurotoxic δ-aminolevulinic acid [93,94]. *CASP3*, a central enzyme in apoptosis, is activated under oxidative stress caused by lead, and may contribute to neurotoxicity and organ damage [95,96].

*HMOX1*, a stress-inducible enzyme involved in heme degradation, is upregulated as a cytoprotective response [97,98]. *APP*, linked to brain metabolism and β-amyloid production, may be altered by lead, promoting neurodegenerative changes [99,100]. Lastly, *NFE2L2* (*NRF2*) regulates antioxidant defenses and may be transiently activated by lead, though chronic exposure may exhaust this protective mechanism [101,102,103]

### 7.2. Pathologies Associated with Lead Exposure

Additionally, it is observed that lead is associated with 4401 pathologies, among which liver cirrhosis (experimental), prostatic neoplasms, and breast neoplasms stand out, characterized by the highest inference scores, suggesting a strong relationship with lead exposure. Other relevant pathologies include hepatocellular carcinoma, autistic disorder, and obesity, all linked to numerous genes involved in complex pathological processes.

The following Table 2 presents the most notable pathologies and their inference scores.

### 7.3. Significant Pathways Enriched in Genes Interacting with Lead

A total of 799 pathways were identified as significantly enriched among the genes interacting with lead, highlighting potential biological processes affected by exposure to this metal. Among the most significant pathways are those related to metabolism, the immune system, and signal transduction, all represented by a substantial number of genes interacting with lead.

The following Table 3 presents the most significant pathways.

Taken together, these findings reveal that lead exerts its toxic effects by disrupting key molecular pathways involved in inflammation, oxidative stress, metal detoxification, and apoptosis. The high number of gene interactions and enriched biological processes highlight the complexity of lead-induced toxicity and its potential role in the development of chronic diseases, including cancer and neurodegenerative disorders [76]. Understanding these molecular mechanisms is essential for identifying therapeutic targets and guiding future preventive strategies.

## 8. Economic and Nutritional Factors in Lead Poisoning

Currently, limited or insufficient economic resources play a key role in lead poisoning, as they hinder access to health education and adequate nutrition, essential factors to prevent such poisoning [20]. Diet and nutritional status are crucial determinants in the absorption of lead, since certain nutritional deficiencies can increase susceptibility to lead toxicity [7].

It is well known that folate is an essential nutrient for the proper neurological development of the fetus during pregnancy. Folic acid supplementation is recommended as a preventive measure to improve folate levels in the body, as this nutrient acts as a buffer against the toxic effects of lead, especially with regard to neural tube defects [69].

Deficiency of certain nutrients, such as iron and calcium, also plays an important role in lead poisoning. Lack of these nutrients increases the absorption of the toxic metal in the body. In particular, iron deficiency has been linked to increased uptake of lead through the digestive tract, which increases the risk of poisoning [3].

Studies have identified a relationship between elevated levels of lead and calcium and selenium deficiencies during pregnancy. Therefore, the intake of calcium and selenium supplements is recommended, and can not only improve nutritional status, but also reduce the risk of complications such as miscarriage [68].

A diet rich in essential minerals such as calcium, iron, zinc, and copper can help reduce lead absorption, thus improving health status and reducing the negative effects of exposure [7]. Furthermore, several studies have found that exposure to lead is associated with an increased risk of depression, although this can be attenuated by regular physical activity, which also contributes to mental health [104].

For instance, a randomized controlled trial conducted in Mexico found that daily calcium supplementation in pregnant women significantly reduced blood lead levels [105]. Similarly, iron supplementation has been shown to decrease gastrointestinal lead uptake in anemic children [106]. These findings underscore the need to implement targeted nutritional interventions in vulnerable populations, particularly in mining regions or urban slums, where lead exposure remains a major concern.

## 9. Blood Lead Levels in Lead Poisoning

Blood lead detection has been one of the main tools for the prevention of lead poisoning. However, measuring blood lead levels alone does not completely prevent the consequences associated with poisoning [39]. This measurement is useful for assessing lead exposure at various points in life, as it allows for monitoring the amount of lead in different tissues of the body [74].

Recent studies have shown a significant decrease in blood lead levels in several regions, such as Latin America and the Caribbean. For example, a study [40] found that the prevalence of children with lead levels above 10 μg/dL is 6.78%, which represents a considerable improvement compared to a previous review [38], which reported a prevalence of 22.08% in children with levels above 10 μg/dL. In Mexico, the National Institute of Public Health reported, in 2023, that 16.8% of children had blood lead levels ≥5.0 μg/dL [107]. Similarly, studies in Ecuador revealed alarmingly high levels, with a mean concentration of 29.4 μg/dL among young children [108]. These findings highlight the urgent need for coordinated, evidence-based public health strategies and sustained policy efforts to reduce lead exposure in children, particularly in vulnerable communities across Latin America.

Despite efforts to reduce lead exposure, in countries such as the United States, non-Hispanic white racial and ethnic groups continue to show higher blood lead levels compared to other groups, although the disparity has decreased. These elevated levels are thought to be largely due to socioeconomic factors [109].

While current policies have contributed to the decrease in lead exposure in younger generations, it is important to remember that older generations were also exposed to much higher levels of lead, with some children exposed to levels five times higher than currently allowed (5 μg/dL). Therefore, it is essential to pay attention to these past generations, who continue to require intervention and monitoring [75].

Monitoring blood lead levels remains a key strategy in assessing exposure, yet disparities persist across regions and populations. Although recent data show a decline in lead levels, especially among children, many communities (particularly in low-resource areas) continue to exceed safety thresholds. Continued surveillance and targeted interventions are necessary, not only for current at-risk populations, but also for older generations previously exposed to high levels of lead.

## 10. Key Findings and Conceptual Summary of Lead Toxicity

**Figure 5:**
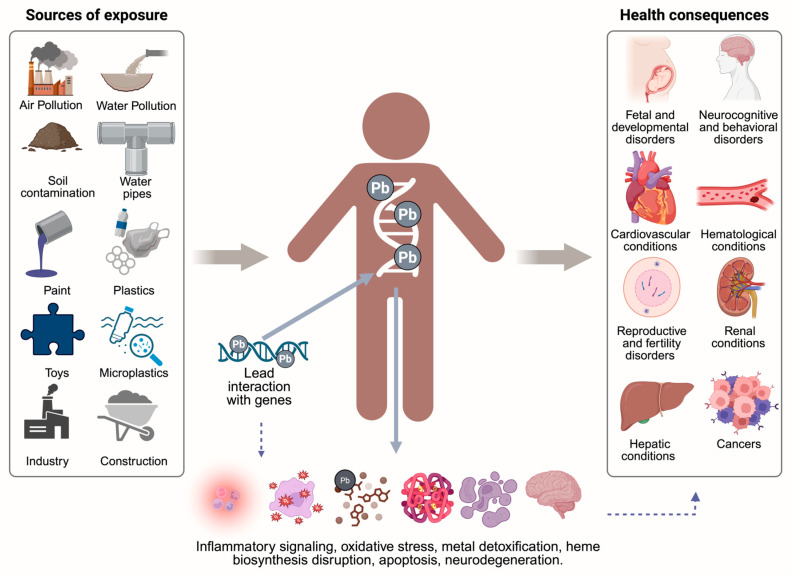
Lead exposure: sources, molecular interactions, and health consequences. Lead exposure sources such as air, water, and soil pollution; industrial activity; construction materials; paint; plastics; microplastics; and contaminated toys, contribute to its accumulation in the body [53,54,55,64]. Once absorbed, lead disrupts gene expression and cellular functions, triggering inflammation, oxidative stress, impaired detoxification, and neurodegeneration, which are linked to developmental, neurological, cardiovascular, reproductive, renal, hepatic, and cancer-related outcomes [78,83,86,94,95,96,97,101,110]. Created in BioRender. Garibaldi, A. (2025) https://BioRender.com/w9f7ya4.

Based on the reviewed evidence and the key findings described throughout this article, Figure 5 summarizes the main sources of lead exposure, its interaction with the human genome, and the downstream activation of pathological molecular mechanisms. This integrative schematic also outlines the major systemic health consequences associated with lead poisoning, offering a comprehensive overview of the biological cascade from exposure to disease.

## 11. Conclusions

Lead poisoning remains a major public health concern across the Americas, particularly in Latin America and the Caribbean, where children, pregnant women, and socioeconomically disadvantaged communities face disproportionate risks. Despite regulatory advances in some countries, persistent exposure through occupational, environmental, and domestic sources continues to threaten vulnerable populations. Emerging sources such as microplastics and electronic waste further complicate the regional scenario. Widespread socioeconomic inequalities, inadequate enforcement of regulations, and aging infrastructure exacerbate exposure and hinder effective prevention.

Molecular and toxicogenomic evidence indicates that lead interferes with key biological processes including inflammation, oxidative stress, metal detoxification, and neurodevelopment, contributing to a wide spectrum of health effects such as cognitive impairment, developmental delays, anemia, kidney and liver damage, reproductive dysfunction, and increased cancer risk. In the Americas, these outcomes disproportionately affect marginalized populations with limited access to healthcare and nutritional resources.

To confront this multifactorial challenge, region-specific strategies are urgently needed. This includes strengthening surveillance systems, updating local reference levels for blood lead, improving regulatory enforcement, promoting public awareness, ensuring access to safe housing and water, and integrating nutritional and environmental interventions into public health programs. Additionally, interdisciplinary research and investment in affected communities are essential to reduce exposure, mitigate long-term consequences, and promote environmental justice and health equity throughout the Americas.

## Figures and Tables

**Table 1:** Impact of lead exposure on maternal and fetal health during pregnancy.

Category	Effects on Pregnant Woman	Effects on Fetus or Newborn	Reference(s)
**General Exposure**	A 3.82-fold increased risk of elevated blood-lead levels	Lead crosses the placenta and reaches the fetus	[3,9,69]
**Toxicokinetics and Mechanisms**	Only ~1% of plasma lead crosses the placenta Lead can be mobilized from maternal bones, especially during the third trimester	Increased fetal exposure in late pregnancy	[9,68,69]
**Maternal Symptoms**	Lethargy, fatigue, abdominal pain	-	[9]
**Pregnancy Complications**	Associated with preeclampsia (based on prior findings)	-	[3]
**Neurotoxicity and Fetal Development**	-	Risk of neurological impairment due to lead neurotoxicity	[9,68,69]
**Perinatal Outcomes**	-	Low birth weight and risk of miscarriage	[66,68,69]
**Environmental Risk Factors**	Recent painting, urban congestion, iron and calcium deficiency	-	[9]
**Maternal–Cord Blood Correlation**	-	Significant correlation between maternal and cord blood-lead levels (rs = 0.63, *p* < 0.001)	[9]

**Table 2:** Pathologies with the Highest Inference Scores Associated with Lead [76].

Disease	Genes	Inference Score
Liver Cirrhosis, Experimental	198	176.43
Prostatic Neoplasms	174	150.46
Breast Neoplasms	158	106.5
Carcinoma, Hepatocellular	153	128.36
Chemical and Drug-Induced Liver Injury	120	11.42
Lung Neoplasms	93	59.95
Stomach Neoplasms	92	80.07
Autistic Disorder	89	98.15
Colorectal Neoplasms	89	82.63
Hypertension	82	16.85
Autism Spectrum Disorder	78	66.49
Disease Progression	77	90.5
Disease Models, Animal	72	43.52
Neoplasm Metastasis	70	46.09
Neoplasm Invasiveness	69	45.73
Carcinoma	66	65.18
Obesity	66	51.11

**Table 3:** Biological Pathways Significantly Enriched in Genes Interacting with Lead [77].

Pathway	Corrected *p*-Value	Annotated Genes
**Metabolism**	2.53 × 10^−187^	489
**Immune System**	9.66 × 10^−180^	474
**Gene Expression**	9.79 × 10^−145^	399
**Signal Transduction**	2.20 × 10^−141^	473
**Protein Metabolism**	1.77 × 10^−129^	357
**Innate Immune System**	8.33 × 10^−121^	309
**Disease**	4.07 × 10^−104^	235
**Developmental Biology**	1.21 × 10^−99^	257
**Hemostasis**	5.93 × 10^−96^	196
**Metabolic Pathways**	1.94 × 10^−83^	258

## Data Availability

No new data were created or analyzed in this study.
